# Evidence of Horizontal Violence in Healthcare Settings: A Narrative Review

**DOI:** 10.3390/nursrep14030123

**Published:** 2024-07-11

**Authors:** Guido Vittorio Travaini, Emma Flutti, Martina Sottocornola, Vittoradolfo Tambone, Alberto Blandino, Gianmarco Di Palma, Francesco De Micco

**Affiliations:** 1 School of Medicine, University of Vita-Salute San Raffaele, 20132 Milan, Italy; travaini.guido@hsr.it (G.V.T.); m.sottocornola@studenti.unisr.it (M.S.); blandino.alberto@hsr.it (A.B.); 2 Department of Human Neuroscience, Sapienza University of Rome, 00185 Rome, Italy; 3 Bioethics and Humanities Research Unit, Campus Bio-Medico University of Rome, 00128 Rome, Italy; v.tambone@unicampus.it (V.T.); g.dipalma@policlinicocampus.it (G.D.P.); f.demicco@policlinicocampus.it (F.D.M.); 4 Campus Bio-Medico University Hospital Foundation, 00128 Rome, Italy; 5 Public Health, Experimental and Forensic Sciences Department, University of Pavia, 27100 Pavia, Italy

**Keywords:** lateral violence, internal violence, interpersonal aggression among colleagues, peer-to-peer violence, co-worker mistreatment, worker-to-worker violence, workplace bullying, medical facilities, clinical settings, hospital

## Abstract

In the professional realm, the healthcare sector stands out as one of the most susceptible to violence. One notable manifestation of this is violence among colleagues, commonly referred to as horizontal violence, which has garnered significant attention in recent times. To delve deeper into this phenomenon across various categories of healthcare professionals, a comprehensive search was conducted on PubMed, Scopus, and CINAHL, resulting in the inclusion of 13 articles. The findings of this narrative review illuminate how horizontal violence can manifest in both physical and psychological forms and how it often becomes normalized among the healthcare professionals who endure it. Particularly vulnerable to such occurrences are recent graduates and those with limited professional experience. Furthermore, it has been observed that horizontal violence has detrimental effects on the well-being of those subjected to it, as well as on the quality of patient care delivered. Considering preventive measures, numerous studies emphasize the pivotal role of effective departmental leadership in fostering a harmonious work environment. Despite the largely underreported nature of this phenomenon, the conclusions drawn in this study advocate for a deeper exploration of the dynamics surrounding horizontal violence, with the goal of devising targeted strategies to mitigate its occurrence.

## 1. Introduction

In the healthcare sector, the workplace can be a stage of violence: the Bureau of Labor Statistics Census of Fatal Occupational Injuries—The Bureau of Labor Statistics (BLS) Census of Fatal Occupational Injuries (CFOI) produces comprehensive, accurate, and timely counts of fatal work injuries. CFOI is a Federal-State cooperative program that has been implemented in all 50 States and the District of Columbia since 1992- in fact, stated that healthcare workers are 16 times more likely to suffer violence than other workers [1]. Among these, especially in Emergency-Urgency Departments, a high probability of victimization is observed [2,3,4], although no type of health worker can be considered completely free from this risk, as also demonstrated by studies concerning psychiatry, radiology, and infectious diseases departments [5,6,7].

Indeed, on the subject of violence in the workplace, the Californian Division of the Occupational Safety and Health Administration [8] has defined three distinct categories of violence, using as a criterion of differentiation the relationship between the active and passive subject of the aggression: (i) violence caused by persons outside the organization; (ii) violence caused by the users of the organization; (iii) violence caused by the operators of the organization itself, such as colleagues or superiors.

Here, we will focus mainly on the third of the above-mentioned cases, also known as horizontal violence (or lateral violence or internal violence and/or type III violence), which is a topic of great interest, given the small number of studies on this point already present in the literature [9,10,11].

In particular, it occurs when a member of the hospital staff uses violence (verbal and/or physical) against a colleague: it could occur anywhere, but, as far as the health sector is concerned, it has been shown to have a high incidence in the United States [12], particularly impacting vulnerable individuals who have less confidence in their professional abilities [12]. However, it appears to be very difficult to calculate an exact estimate of those who have experienced it, given the high dark number of violent episodes in the healthcare sector [3].

In any case, horizontal violence produces a hostile environment and extremely damaging consequences for healthcare workers, so the latter suffer physically and psychologically, developing, for example, depressive symptoms, anxiety, and sleep disorders [13]. In addition, a reduction in productivity has been observed in those who have undergone it, as well as a decrease in the quality of care offered to patients in the relevant wards [9,10,11,12,13,14]. Therefore, it is imperative to delve deeper into this phenomenon, which is considered in this review aiming to broaden the scope of investigation beyond the nursing population, which is commonly the focus in existing literature reviews (e.g., [15,16,17]).

Indeed, this study represents the first comprehensive literature review conducted to provide a preliminary depiction of horizontal violence occurring among colleagues within the healthcare environment, encompassing all professional categories.

The decision to prioritize the examination of horizontal violence in healthcare settings is thus underscored by its significance within workplace dynamics, its implications for both employee welfare and the quality of patient care, and the comparatively lower frequency with which it is reported compared to other forms of violence. Consequently, concentrating on type III violence not only addresses a notable gap in existing literature but also advances efforts towards a more comprehensive understanding and prevention of this phenomenon.

## 2. Materials and Methods

Literature review: PubMed, Scopus and CINAHL were searched for peer-reviewed studies on horizontal violence among healthcare workers. The selection was based on these databases being regarded as foremost and optimal for research within the realm of health sciences and professions as well as they represent search engines subject to regular content updates and user-friendly accessibility. Moreover, our selection was informed by their prominent use in healthcare violence research: PubMed offers a comprehensive repository of biomedical literature, including a dedicated section on criminology and forensic sciences; Scopus is recognized for indexing high-quality scholarly content across medicine, social sciences, psychology, and criminology; CINAHL is widely utilized in health sciences, particularly nursing, which is pertinent to incidents of healthcare violence. We opted against including Web of Science due to its primary focus on natural sciences, limited coverage of health sciences topics, restricted content access, and less frequent updates, potentially impacting the comprehensiveness of our review. The database search was carried out using the terms: ‘horizontal’, ‘lateral’, ‘internal’, ‘worker-to-worker’, ‘violence’, ‘mistreatment’, ‘hospital’, and ‘healthcare’. The search was limited to the period 2013–2023 to focus on current studies on the research phenomena. The final search was carried out on 19 December 2023. From the initial database search, 67 publications were deemed potentially relevant. A secondary supplementary literature search involved a manual search of the reference lists of the retrieved papers by two investigators (E.F. and M.S.). A combined total of 71 were identified as of interest to this narrative review. After removing duplicates, 50 titles and abstracts of each study were identified and then screened for eligibility. Thirty-two papers survived this screening process and were then assessed to determine whether the literature met the inclusion criteria based on a full-text reading. Of these, one was unavailable even after contacting the corresponding author. All remaining papers were downloaded and revised. Thirteen articles were ultimately included in the narrative review, as listed in Table 1.

Determining eligibility: each study included in this review evaluates the topic of horizontal violence in healthcare settings. Only original research studies, conducted on healthcare workers, have been taken into consideration. We omitted papers focused on related topics. Non-research papers or review papers were excluded as well as studies not written in either Italian or English.

## 3. Results

### 3.1. Phenomenology

#### 3.1.1. Typology of Horizontal Violence

Horizontal violence between health professionals manifests itself through different channels. Most of the studies considered agree in identifying psychological, verbal, and physical violence as the most representative categories. Ebrahimi, Hassankhani, Negarandeh, Jeffrey, and Azizi [18], through a conventional content analysis approach applied to 18 conducted interviews, were able to identify the following four categories or themes: ‘Psychological violence’, ‘Verbal violence’, ‘Physical violence’ and ‘Source of violence’. Sub-categories or sub-themes were also identified, totaling 10: ‘Isolating the person’ and ‘Destroying self-esteem’ as main manifestations of psychological violence; ‘Verbal harassment’ and ‘Biased reporting’ for Verbal violence; ‘Physical threat’ and ‘Taking advantage’ for Physical violence; and ‘Discrimination’, ‘Wrongly judging’ ‘Indebted to supporter’, and ‘Inadequate support’ as main Sources of violence. It is important to report that participants of this study made no mention to sexual violence, since sexual issues are considered taboo in Iranian culture. In another study [20], the respondents recognized ‘physical’, ‘verbal’, and ‘emotional’ violence as the main categories of violence encountered. Instances of physical violence encompass actions such as pushing, slapping, kicking, punching, throwing items, and property destruction. Verbal and emotional violence involve behaviors such as cursing, insulting, patronizing, verbally threatening, bullying, and body-shaming. Many of the participants asserted that verbal and/or emotional violence constitutes the most prevalent form. In Rauman’s study [23], the typical behaviors and attitudes mentioned in the participants’ accounts included yelling, belittling, denial of assistance, ignoring, withholding information, and the phenomena of nurses mistreating their younger colleagues, being left to fend for themselves, and being set up to fail. Another study, highlights that tools of horizontal violence primarily include verbal aggression, as reported by recently graduated nurses interviewed [25]. These nurses describe stressful situations during handovers, impertinent tones in explanations, non-constructive criticism, and threats from senior nursing staff, including the risk of negative evaluations and dismissals. Additionally, other forms of verbal violence, such as personal insults and offensive jokes about appearance and sexual orientation, indifference in all its facets (e.g., avoiding, excluding, ignoring), and not listening to the needs and requests, are mentioned, often occurring in front of others. Ignoring opinions and spreading of gossip were the most reported negative behaviors also found by Peng et al. [21].

#### 3.1.2. Healthcare Professionals’ Victims

The selected studies focused mainly on investigating the phenomenon of horizontal violence between nurses. In fact, only four of them considered a sample of other hospital professionals [19,26,27,29]. More specifically, the study by Volz et al. [29], examined the category of physicians in emergency doctors, while the studies by Hamblin [19], Taylor [29], and Taylor and Taylor [27] addressed hospital staff. This revealed that healthcare professionals overall, even if not nurses, are not immune to horizontal violence. Considering the studies focused on the nursing population, a recurring topic was identified in the strong victimization of newly graduated nurses. Thus, Read and Laschinger [24] decided to consider a sample consisting only of the latter (n = 342), hence identifying three different forms of workplace mistreatment: co-worker incivility, supervisor incivility, and bullying. Similarly, Ebrahimi et al. [18], based on a sample of 18 experienced nurses with an average of 11.27 years of seniority (14 nurses, 2 head nurses, and 2 supervisors), focused on the different forms and sources of horizontal violence against newly graduated nurses. Furthermore, Reynolds, Kelly, and Singh-Carlson [30], contrasting Purpora, Blegen, and Stotts [22], observed that, when examining a sample of 63 nurses in the neonatology department with an average seniority of 24.69 years (13 staff nurses, 6 charge nurses, 6 clinical coordinators, 10 managers, 8 directors, and 20 others), more years of experience corresponded to a lower risk of exposure to horizontal violence. However, the study by Rosi et al. [25], which included a sample of 21 nurses from different departments with an average working experience between eight months and three years, revealed that although newly graduated nurses are often the target of horizontal violence (regardless of their age), they have little knowledge of what horizontal violence is. In fact, those surveyed who have been victims were rarely able to define this phenomenon and the seriousness of its consequences.

#### 3.1.3. Institutionalization

Selected studies have indicated that horizontal violence in healthcare tends to be normalized or downplayed [20,23,25,29]. In one of these studies, findings revealed that nurses working in psychiatric settings often perceive violence as ‘part of the job’ and a common occurrence, normalizing workplace violence [20]. Specifically, an analysis of interviews with 12 nurses identified ‘Violence as a norm in the psychiatric setting’ as a recurring theme, with 83% of participants using terms like ‘common’, ‘normal’, or ‘part of the job’ when describing their experiences with workplace violence. In another study conducted by Rauman [23], the participants’ expression ‘I always understood their position’ provided evidence of the acceptance and normalization of being too busy to offer assistance or mentorship within the hospital environment. Furthermore, the statement ‘kind of leave you to your own devices’ shed light on the expectation for nurses to operate independently. Rosi et al. [25] reported that most respondents, when interviewed, minimized episodes of violence. They either justified or emphasized positive aspects, perceiving the phenomenon as generational. Additionally, by internalizing or attributing blame, newly graduated nurses struggled to recognize these behaviors as forms of horizontal violence. A similar result was also found by Taylor [26], who observed that behaviors linked to horizontal violence were tolerated. It seemed that nurses and other staff members regarded them as inherent to the job, downplaying the lasting effects on communication, teamwork, and safety. Dealing with conflicts among colleagues was not a priority for the nurses in the sample, with some expressing indifference towards establishing harmonious relationships with their coworkers.

### 3.2. Negative Impact

#### 3.2.1. Quality of Care and Professional Performance

Five papers reported findings relating to the impact of horizontal or lateral violence on the quality of care provided by health workers. One of these aimed at understanding the horizontal violence exercised against newly graduated nurses, interviewing 18 experienced Iranian nurses [18]. The results showed that instances of mistreatment in the workplace damage the personality of nurses and the ethical atmosphere of the organization, endangering the safety of patients, who become victims of a destabilized care environment rendered ineffective by the violent behavior of their caregivers. Purpora et al. [22], in their study, found that horizontal violence was inversely related to peer relations and quality of care (r = −0.469; *p* < 0.01): as horizontal violence increased, the quality of care decreased. The same study reported that only horizontal violence and clinical area predicted the quality of patient care, as well as illustrating that it occurs in most nurses, regardless of their expertise or their personal characteristics. A similar result was also found in the study of Reynolds et al. [30], which demonstrated a relationship between horizontal violence and negative patient outcomes or near misses through the analysis of an online survey completed by 63 nurses. P-tests revealed a significant difference in the means among respondents who reported that the forms of horizontal violence they had witnessed or experienced in the last 12 months had resulted in a negative patient outcome or near miss (M = 28.16, SD = 3.88) and those who did not report it (M = 22.41, SD = 6.65, t(58) = 2.86, *p* = 0.005). A study that analyzed the perspectives of 15 nurses regarding mistreatment in the workplace identified three recurring themes, respectively divided into categories and subcategories [28]. Of these, the theme ‘Inaction despite injury’ encompasses the category ‘Negative personal and professional impacts’ which is replicated in the subcategories ‘Decreased commitment’ and ‘Decreased job motivation’. Indeed, the interviewed nurses reported the negative impact that repeated mistreatment over time has on their personal lives, emotions (e.g., losing self-confidence), motivation to work, and commitment to the nursing profession (e.g., considering changing job). Lastly, Volz et al. [29] aimed to investigate the prevalence of horizontal violence among emergency department attending physicians, residents, and mid-level providers. They found that less than 10% of respondents (out of 91) to their online surveys reported that horizontal violence had affected their personal health, led them to think about quitting their job, or made them feel unsafe in their work environment. Nearly a quarter (22.2%) of respondents reported that they could remember a specific time in the preceding 12 months when it had negatively impacted care for their patients.

#### 3.2.2. Psychological Aspects

In a study conducted by Lim et al. [20], most respondents agreed that short-term consequences, such as feelings of sadness, fear, anxiety, sleep difficulties, and stress, are brief. Conversely, most participants maintained that they did not experience any lasting negative effects from workplace violence. However, a small number of participants acknowledged enduring impacts, such as diminished motivation, mental fatigue, and decreased self-confidence. Another study conducted by Rauman [23] observed that participants reported facing stress and frustration in the healthcare environment, with many nurses experiencing burnout under such conditions. Burnout was often attributed to the hospital setting and the working conditions surrounding nurses, leading to strained relationships among registered nurse peers. According to the interviewees in the study conducted by Rosi et al. [25], horizontal violence was shown to affect the emotional, physical, and social well-being of new graduate nurses. This impact resulted in reduced enthusiasm and motivation and heightened feelings of failure and personal defeat, ultimately leading to voluntary resignations and an increase in shift turnovers. Similarly, Read and Laschinger [24] discovered a significant relationship between workplace mistreatment and mental and physical health. Poor physical health was linked to higher levels of bullying (0.39), supervisor incivility (0.33), and coworker incivility (0.28). Poor mental health was also associated with increased levels of bullying (0.32), supervisor incivility (0.28), and coworker incivility (0.25). Specifically, emotional exhaustion was related to bullying (0.46), coworker incivility (0.31), and supervisor incivility (0.35).

### 3.3. Structural Dimension

#### 3.3.1. Role of Leadership

Four of the selected studies [21,24,25,28] showed how efficient leadership models can contain the phenomenon of horizontal violence. Peng et al. [21], demonstrated, by means of a multivariate logistic regression analysis, that the risk of horizontal violence is reduced for nurses whose head nurse presents a more caring attitude. Indeed, in another study, it emerged how the sense of bewilderment resulting from being ‘left to one’s devices’ is itself perceived as a form of horizontal violence [25]. Moreover, Vagharseyyedin [28] highlighted how important it is, with a view to preventing horizontal violence phenomena, to select hospital managers who present a marked emotional intelligence and who work strategically to counter the phenomenon. Similarly, Read [24] emphasized how the perception of an authentic leadership style in supervisors is inversely related with the perception of workplace mistreatment episodes and promotes job satisfaction, especially among new graduates.

#### 3.3.2. Contextual Variables

A further thematic category that emerged from the analysis of the sample of included articles was the role played by contextual factors [19,23,24,27]. A study that aimed to identify the common catalysts of worker-to-worker violence and incivility in hospitals found that most of the incidents reported by employees did not involve physical violence [19]. Two recurring themes emerged from the sample of complaints analyzed (n = 141): ‘Work Behavior’ and ‘Work Organization’. Incidents in the ‘Work Organization’ category involved conflicts or aggression resulting from non-compliance with protocol (n = 16), patient assignment (n = 16), limited resources (n = 7), and high workload (n = 7). This study emphasized the importance of circumstances and understanding—situational catalysts—of work procedures rather than the actual performance of work tasks. Rauman [23] interviewed 17 nurses with the aim of assessing how the hospital was related to horizontal violence. Text analysis showed how contextual variables were influential in regulating relationships between colleagues. In fact, the participants pointed out that starting a conflict seems not necessarily related to the involvement of a single nurse per se so much as to the atmosphere of nursing practice. Conflictuality appears widespread within the professional experience and related to the social organization of nursing in the hospital, which has been described as characterized by the promotion of individualism and competition among fellow nurses. Expectations on the active participation of nurses in broader organizational processes to ensure productivity and efficiency in the hospital environment, such as the implementation of lean processes and hospital policy, were identified by the participants as elements of interference with nursing practice as well as an overload of activities to cope with. This complex health care system affects the effective functioning of the health organization and compromises the quality of patient care. Another study conducted by Taylor [26] found, as a main theme related to horizontal violence, ‘Organizational Chaos’. The 120 interviewed hospital staff described not having access to the equipment or not finding drugs and supplements readily available for use. Nurses pointed out the following as a significant stressor and potential risk to nurse–physician relationships: clustered admissions and discharges which create a chaotic environment where nurses handled multiple discharges while new admissions arrived. Another critical element was inadequate transport (delayed or unavailable), which caused delays in patient treatment. The perception of the disproportion of shift work also had a negative effect on relations between nursing colleagues. Respondents reported that faced with such structural deficiencies, it seemed that there was no other solution but to circumvent the problem or, as often happened, to take it out on those perceived to be behind the problem (transport, pharmaceuticals, or central supply).

#### 3.3.3. Dark Number

In two of the selected studies [20,25], it was emphasized that the phenomenon of horizontal violence between health care employees is characterized by the so-called dark number. Taylor [26] pointed out how, in the human resources department of the healthcare setting, there are very few reports of violent behavior committed by other employees. In addition, Lim et al. [20] underlined that formalizing a complaint tends to occur only when violent incidents result in actual injuries, whereas verbal violence is almost never reported.

## 4. Discussion

The detailed analysis of the results collected through the included studies provides a comprehensive overview of the impacts of horizontal violence in the healthcare context, highlighting several key themes.

Horizontal violence among health professionals takes shape as a societal scourge characterized by a high degree of intolerability. In the recent study conducted by Peng et al. [21], 59.1% out of 1148 participants admit to experiencing horizontal violence. In the context of the study by Purpora et al. [22], 79.4% of participants claim to have experienced it at least once in the six months preceding the investigation. The phenomenon manifests through different channels. Most studies agree on identifying psychological, verbal, and physical violence as the most representative categories. Sexual violence does not fall within the previously mentioned categories. This fact can be interpreted as a result of cultural taboos (e.g., it is not common in Iranian culture to openly discuss issues related to the sexual sphere) or as indicative of the high percentage of unreported cases that characterizes the general population. Additionally, it is important to note that recognizing an act as constituting sexual violence is not always immediate (e.g., the Italian legislature has adopted a comprehensive definition that encompasses any act capable of compromising the sexual freedom of the individual, without the necessity of complete sexual intercourse taking place). It is imperative to comprehensively address these issues to construct conceptual frameworks that facilitate the understanding of this multifaceted problem.

The studies reviewed underscore the pervasive nature of horizontal violence, revealing that it extends beyond nurses to include other healthcare professionals. While most of the research focused on nurses, investigations involving physicians and hospital staff indicate that healthcare professionals at large are susceptible to horizontal violence. Notably, newly graduated nurses emerged as a particularly vulnerable group, with several studies highlighting their heightened victimization. Newly graduated nurses were subjected to mistreatment, encompassing co-worker incivility, supervisor incivility, and bullying. The research emphasizes the need for tailored interventions and support systems to address the specific challenges faced by this demographic.

In the included studies, a notable prevalence of females among the victims is evident. Hamblin et al. [19] brought to light that 81% of the documented cases of mistreatment involved female-gendered employees, whereas Volz et al. [29] noted that women are more frequently subjected to verbal abuse, such as being shouted at. However, it is crucial to note that the predominant composition of female participants, especially among nurses, in the included studies poses challenges in making definitive assessments regarding gender as a variable.

Studies agree on the negative influence of horizontal violence on the quality of care provided by healthcare workers. The inverse correlation between horizontal violence and peer relations indicates that a workplace disturbed by conflicts and aggressions results in a decrease in the quality of care. This raises significant concerns for patient safety, as the ethical atmosphere of the organization is compromised. Furthermore, the negative psychological consequences, although initially perceived as short-term, can evolve into lasting impacts on mental and physical health. Decreased motivation, mental fatigue, and reduced self-confidence are issues that require attention and targeted interventions.

Efficient leadership emerges as a crucial factor in containing horizontal violence within healthcare settings. Studies consistently show that a caring attitude from head nurses reduces the risk of horizontal violence, and authentic leadership styles, characterized by emotional intelligence, significantly contribute to preventing workplace mistreatment. This underscores the importance of selecting and nurturing leaders who can strategically counteract the phenomenon.

Contextual variables emerge as critical factors influencing the dynamics of horizontal violence. The lack of resources, high workloads, and organizational dysfunctions are identified as significant catalysts for violence among colleagues in the included studies. This aligns with the systemic factors identified by Lim and Bernstein [31]: work pressure, involving increased demands for productivity, cost containment, and a rigidly structured organizational hierarchy, anxiety stemming from potential legal disputes, disparities among workers in terms of autonomy, authority, empowerment, roles, and values, and persistent instability marked by frequent staff changes and shift rotations. These results emphasize the importance of addressing organizational issues to prevent and manage horizontal violence.

Two studies [20,26] draw attention to the concept of the ‘dark number’ in horizontal violence—incidents that go unreported or underreported for several reasons, but mainly due to the lack of changes after reporting. This phenomenon is particularly evident in verbal violence, where formal complaints are rare unless incidents result in physical injuries. The underreporting suggests that the true extent of horizontal violence may be underestimated, emphasizing the need for enhanced reporting mechanisms and a supportive environment for victims. Moreover, the tendency to internalize and minimize horizontal violence among healthcare workers raises concerns about the distorted perception of violence as ‘part of the job’. This attitude could hinder efforts to address the problem at its root and implement effective interventions.

### 4.1. Practical Implications

The identification of these themes holds significant practical relevance. According to the Joint Commission International—The Joint Commission International (JCI) is the recognized global leader in health care accreditation—it is necessary for healthcare organizations to develop prevention and protection measures, train healthcare workers in recognizing and managing aggressive behaviors, ensure support for victims, promote event reporting, and conduct detailed event analysis to determine contributing factors, including workplace conditions, in order to identify priority intervention situations [32].

The American Hospital Association identified that in 2020, almost 60% of surveyed hospitals implemented workplace violence prevention initiatives [33]. An Italian Cross-Sectional Survey showed that female gender was associated with a 2.6-times-higher risk for the presence of aggression, while working as a nurse was linked to an approximately fourfold-increased risk of encountering aggression. Additionally, the risk of aggression increased by 5% for each year of work experience [34].

Therefore, a healthcare organization permeated by a safety culture should implement tools to improve the safety of its workers through structured and organized interventions that protect employees and patients from any form of violent behavior. In particular, training programs are essential to ensure that all workers are aware of risks and know how to protect themselves and others through the application of specific procedures. The goal is to provide training on specific risks associated with the activity performed, including methods for recognizing danger signs or situations that may lead to aggression and methodologies for managing aggressive and violent patients. Training initiatives should prioritize interactive teaching methodologies with practical exercises and simulations to enable all healthcare workers to recognize high-risk situations and adopt the most appropriate safety measures.

Implementing organizational policies to enhance the work climate, provide ample resources, and foster a culture of respect and collaboration is imperative. Training interventions should not only concentrate on preventing violence but also emphasize effective conflict management and mental health education. Considering how promoting the psychological well-being of healthcare professionals could contribute to reducing the incidence of horizontal violence. Stress management strategies and psychological support could be integrated as an integral part of organizational initiatives. The findings emphasize the need for comprehensive strategies to address horizontal violence across diverse healthcare professionals. This involves implementing targeted interventions for newly graduated nurses, establishing leadership development programs, and initiating initiatives to promote reporting while addressing the dark number phenomenon. Cultivating a culture of respect and support within healthcare organizations is crucial to mitigating the impact of horizontal violence on both individuals and the quality of patient care. Additionally, interventions should extend beyond reactive measures to encompass proactive strategies, emphasizing prevention and the cultivation of supportive workplace cultures. Through collaborative efforts and a multifaceted approach, healthcare organizations can strive to eradicate horizontal violence and foster a healthier, more conducive work environment for all professionals.

### 4.2. Potential Future Directions

Future research endeavors should aim to delve deeper into the dynamics of horizontal violence within specific healthcare settings. This focused approach can illuminate nuances that may be overlooked in broader studies and enable the development of more targeted and context-specific interventions. Researchers should consider exploring the interplay of variables unique to distinct healthcare contexts, allowing for a more nuanced understanding of the factors contributing to horizontal violence. Additionally, efforts to investigate the longitudinal aspects of horizontal violence can provide valuable insights into the persistence and evolution of these behaviors over time. Longitudinal studies may help uncover patterns, contributing factors, and potential shifts in the prevalence and nature of horizontal violence within the dynamic healthcare landscape. Furthermore, examining the effectiveness of existing interventions and preventive measures could guide the refinement and development of evidence-based strategies. Assessing the long-term impact of interventions on reducing horizontal violence and improving workplace dynamics is essential for fostering sustainable positive change. In conclusion, while the present findings offer valuable contributions to understanding horizontal violence in healthcare, future research endeavors should navigate these acknowledged limitations and pursue avenues that deepen our comprehension of this complex phenomenon in specific and nuanced contexts.

### 4.3. Study Limitations

It is crucial to recognize that some studies included in this analysis may have inherent limitations, ranging from issues related to sample representativeness to context specificity. While the findings provide valuable insights, caution is warranted in generalizing them across diverse healthcare environments. Moreover, although a narrative review was chosen to provide a descriptive and comprehensive overview of the topic without restricting the search to any specific factors or variables, it is important to highlight the limitations inherent in such a methodological approach. Foremost among these is the risk of bias in study selection, which often relies on the subjective judgment of the authors. Similarly, there is a risk of bias in the analysis of included studies, which may be influenced by the authors’ prior knowledge and expertise in the field. Furthermore, it is noteworthy that the sample size of 13 articles may be deemed inadequate for deriving broadly generalizable conclusions. Moreover, the study involved seven authors. The decision to engage a substantial number of authors was justified by the diverse expertise and skills each contributor brought to the study, thereby enriching the analysis and discussion of the topic from various perspectives. This approach ensured a comprehensive and well-founded exploration of the issues addressed in the article. As a result, our study may lack completeness, as it may not have identified all pertinent studies on the topic, and it may also suffer from limited generalizability due to a less rigorous methodology and a non-systematic synthesis of results. Nevertheless, despite these limitations, the article makes a significant contribution to the understanding of the topic within its defined scope.

## 5. Conclusions

The pervasive issue of horizontal violence in healthcare demands a multifaceted response. Addressing the root causes within organizational structures and fostering a culture of respect and support among healthcare professionals are paramount. To truly make strides in reducing the incidence of this phenomenon and elevating the quality of care, it is imperative to embark on collaborative efforts and implement targeted interventions. This calls for a united commitment from healthcare organizations, professionals, and stakeholders to create a transformative and healthier work environment that prioritizes the well-being of individuals and, by extension, the patients they serve.

## Figures and Tables

**Table 1:** Studies included in the review.

References	Study Aim	Study Design and Methods	Sample	Main Outcomes
[18] Ebrahimi, Hassankhani, Negarandeh, Jeffrey and Azizi (2017), Iran	To examine the experience of Iranian experienced nurses’ use of lateral and horizontal violence against new graduated nurses.	Qualitative. Interviews (unstructured general questions, followed by semi-structured questions).	Total of 18 experienced nurses. Recruited via hospital.	Various types of violence are used against newly graduated nurses in the workplace: these harm individual nurses, the quality of care, and the ethical climate of the healthcare facilities.
[19] Hamblin et al. (2015), USA	To identify common catalysts of worker-to-worker violence and incivility in hospital settings.	Qualitative—retrospective descriptive study. Content analysis of employee incidents reports.	Total of 141 worker-to-worker violence incident reports. Collected via hospital system.	Incidents of worker-to-worker violence derived from dissatisfaction with co-workers’ performance or organizational practices.
[20] Lim, Idris, Abdullah and Omar (2023), Brunei	To explore nurses’ experience with workplace violence and the impact of violence on nurses in the psychiatric setting.	Qualitative explorative study. Thematic analysis of online interviews.	Total of 12 nurses. Recruited via hospital, snowballing.	Workplace violence in the psychiatric setting is normalized, has a negative short- and long-term impact, and is rarely reported.
[21] Peng et al. (2021), China	To analyse the prevalence of nurse-to-nurse horizontal violence in Chinese hospitals and examine the effects of head nurses’ caring and nurses’ group behavior on it.	Quantitative. Online questionnaire.	Total of 1942 questionnaires. Recruited via hospital online service.	Total of 59.1% of respondents had experienced horizontal violence, covert negative behaviors were more reported, head nurses’ caring and nurses’ group behavior emerged as protective factors.
[22] Purpora, Blegen and Stotts (2015), USA	To investigate the relationship between horizontal violence, peer relation, quality of care, and errors and adverse events.	Quantitative. Online survey.	Total of 175 hospital staff nurses. Recruited via a paper-based or online survey.	Horizontal violence is inversely related to peer relations and quality of care and positively related to errors and adverse events.
[23] Rauman (2023), Canada	To examine how nursing is socially organized within the hospital setting and how this is linked to conflict in working relationships.	Qualitative. Interviews, text analysis and institutional ethnography.	Total of 17 registered nurses. Recruited via hospital.	Conflict in nursing work environment has become institutionalized. The contextual variables surrounding nursing practice are very influential with respect to how nurses relate to each other.
[24] Read and Laschinger (2013), Canada	To explore correlates of new graduate nurses’ experiences of workplace mistreatment.	Quantitative. Online survey.	Total of 342 new graduate nurses. Recruited via post.	Bullying and workplace incivility have a negative impact on the health and work of newly graduated nurses, who may even end up leaving the profession due to the negative effects of these experiences. By the way, it can be prevented by genuine leadership and stimulating working environments.
[22] Reynolds, Kelly and Singh-Carlson (2014), USA	To determine the frequency of horizontal violence in a perinatal service and its effect on patient outcomes.	Quantitative. Online survey.	Total of 63 nurses. Recruited via university online forum.	Labor and delivery wards are more exposed to horizontal violence than other units included in perinatal services and nurses with fewer years of experience are more likely to be victims of it. There is also a relationship between this type of violence and bad patient outcomes.
[25] Rosi, Contiguglia, Millama and Rancati (2020), Italy	To evaluate the direct and indirect experiences of horizontal violence in newly graduated nurses.	Qualitative. Interviews (first open general question, followed by semi-structured questions).	Total of 21 newly graduated nurses. Recruited via email.	Horizontal violence is rarely recognized by newly graduated nurses, even when they directly or indirectly experienced it.
[26] Taylor (2016), USA	To explore horizontal violence and nurses’ perceptions of the phenomenon.	Qualitative inquiry. Over observation, document review, and nonstructured and semi-structured interviews.	Total of 120 hospital staff, of which 31 participated in semi-structured interviews. Recruited via hospital.	Minimization and non-recognition of behavior, fear as a reporting inhibitor, avoidance and isolation as coping strategies, lack of support and respect, and organizational chaos emerged as major themes of horizontal violence between nurses.
[27] Taylor and Taylor (2017), USA	To suggest an alternative approach to improve recognition of horizontal violence using enactor types identified in a study exploring nurses’ perceptions of horizontal violence.	Qualitative descriptive study. Observation, document review and semi-structured interviews.	Total of 120 participants: 80 nurses, 22 patient care assistants, 14 unit secretaries, 2 nurse educators, and 2 nurse managers (31 participants were interviewed). Recruited via hospital.	Nurse participants identified violent behaviours by describing the attributes of the enactor and the situation, more rarely referring to hospital policies or nursing literature. Three enactor types were disaggregated from the data: the pathological bully, the self-justified bully, and the unprofessional co-worker.
[28] Vagharseyyedin (2016), Iran	To report the perspectives of a sample of Iranian nurses concerning workplace mistreatment.	Qualitative. Semi-structured interviews.	Total of 15 nurses. Recruited via hospital.	Workplace mistreatment negatively influences nurses and services within hospitals. For this reason, organizations must prevent mistreatment wherever possible and when it occurs, take steps to reduce its prevalence.
[29] Volz, Fringer, Walters and Kowalenko (2017), USA	To investigate the prevalence of horizontal violence among emergency department attending physicians, residents, and mid-level providers.	Quantitative. Online survey.	Total of 91 participants: physicians, residents, and mid-level providers. Recruited via mail.	Horizontal violence and its impact on staff and patients are prevalent among emergency medicine attending physicians, residents and mid-level providers.

## Data Availability

Not applicable.

## Public Involvement Statement

No public involvement in any aspect of this research.

## Guidelines and Standards Statement

This manuscript was drafted against the Enhancing transparency in reporting the synthesis of qualitative research (ENTREQ) for narrative reviews [35].
